# Dicer suppresses the malignant phenotype in VHL-deficient clear cell renal cell carcinoma by inhibiting HIF-2α

**DOI:** 10.18632/oncotarget.7807

**Published:** 2016-03-01

**Authors:** Yang Fan, Hongzhao Li, Xin Ma, Yu Gao, Xu Bao, Qingshan Du, Minghui Ma, Kan Liu, Yuanxin Yao, Qingbo Huang, Yu Zhang, Xu Zhang

**Affiliations:** ^1^ Department of Urology, State Key Laboratory of Kidney Diseases, Chinese People's Liberation Army General Hospital, PLA Medical School, Beijing, People's Republic of China; ^2^ Medical School, Nankai University, Tianjin, People's Republic of China

**Keywords:** clear cell renal cell carcinoma, von Hippel–Lindau, hypoxia-inducible factor, dicer, microRNA

## Abstract

Both the von Hippel-Lindau (VHL)/hypoxia-inducible factor (HIF) pathway and microRNA (miRNA) regulation are important mechanisms underlying the development and progression of clear cell renal cell carcinoma (ccRCC). Here we demonstrate that VHL deficiency leads to downregulation of Dicer and, in turn, defects in the miRNA biogenesis machinery in ccRCCs. Dicer inhibited expression of HIF-2α, which was a direct target of Dicer-dependent miR-182-5p in VHL-deficient ccRCCs. Ectopic Dicer expression in VHL-deficient ccRCCs suppressed tumor growth and angiogenesis by inhibiting HIF-2α both *in vitro* and *in vivo*. Reduced Dicer mRNA levels served as an independent prognostic factor for poor survival in patients with VHL-deficient ccRCC. Our results indicate that downregulation of Dicer in VHL-deficient ccRCCs contributes to high levels of HIF-2α and a malignant phenotype, which suggests Dicer could be a useful therapeutic target for managing this disease.

## INTRODUCTION

Clear cell renal cell carcinoma (ccRCC) is the most malignant form of kidney cancer and accounts for approximately 80% of all RCC histologic subtypes [1]. It is a highly aggressive disease, with metastasis already present in 30% of patients at initial diagnosis [2]. Surgery remains the best treatment for localized disease, but advanced ccRCC has a poor prognosis due to its resistance to chemotherapy and radiotherapy. Although novel targeted therapies with relatively good clinical outcomes have been developed, inconsistent responses and the risk of adverse effects limit the use of these drugs [3]. A better understanding of molecular genetics of ccRCC may help identify novel biomarkers for early diagnosis and more effective treatment.

Most ccRCC tumors involve mutation or silencing of the von Hippel-Lindau tumor suppressor (pVHL) gene (VHL) (75%–85%) [4]. The pVHL protein is a critical component of an E3 ubiquitin ligase complex that targets hypoxia-inducible factor (HIF) and affects polyubiquitination and proteasomal degradation [5]. The HIF subunits (HIF-1α and HIF-2α) are transcription factors that mediate cellular adaptation to hypoxia and activate specific genes to alter metabolism, proliferation, angiogenesis, and extracellular matrix remodeling [6]. Under normoxia, HIFs are targeted and ubiquitinated by pVHL, leading to their degradation by cellular proteasomes. However, the absence of functional pVHL in ccRCC impairs HIF degradation and leads to abnormal activation of downstream oncogenes, which contributes to ccRCC development [7]. While HIF-1α and HIF-2α have a number of overlapping properties, they also have distinct roles and regulatory mechanisms, and HIF-2α is the primary oncogenic driver in ccRCC [6, 8].

MicroRNAs (miRNAs) are small noncoding RNAs 18–24 nucleotides in length that regulate gene expression [9]. Aberrant miRNA expression has been observed in various tumor types [10], and an overall reduction in miRNA expression is a feature of many cancers [11]. Recent studies examining alterations in miRNA expression in renal cancer as compared to normal tissues suggest that miRNAs affect ccRCC progression [12, 13]. Although overall miRNA downregulation has been reported in ccRCC, the mechanisms behind this downregulation are not fully understood. The enzyme Dicer processes precursor miRNA into the mature molecule within the cytoplasm [9]. Given that VHL-dependent miRNA regulation has been reported [14] and that Dicer is downregulated in ccRCC [15], alterations in the *VHL* gene might affect miRNA biogenesis and thus contribute to ccRCC progression.

In this study, we examined whether VHL deficiency affects Dicer levels in ccRCCs. We also investigated the effects of changes in Dicer activity and Dicer-dependent miRNA levels on HIF-2α expression. Finally, we determined whether ectopic Dicer expression alters HIF- 2α activity and malignancy, and we also determined the prognostic value of Dicer in VHL-deficient ccRCCs.

## RESULTS

### Dicer is downregulated in ccRCC in a VHL-dependent manner

**Figure 1:**
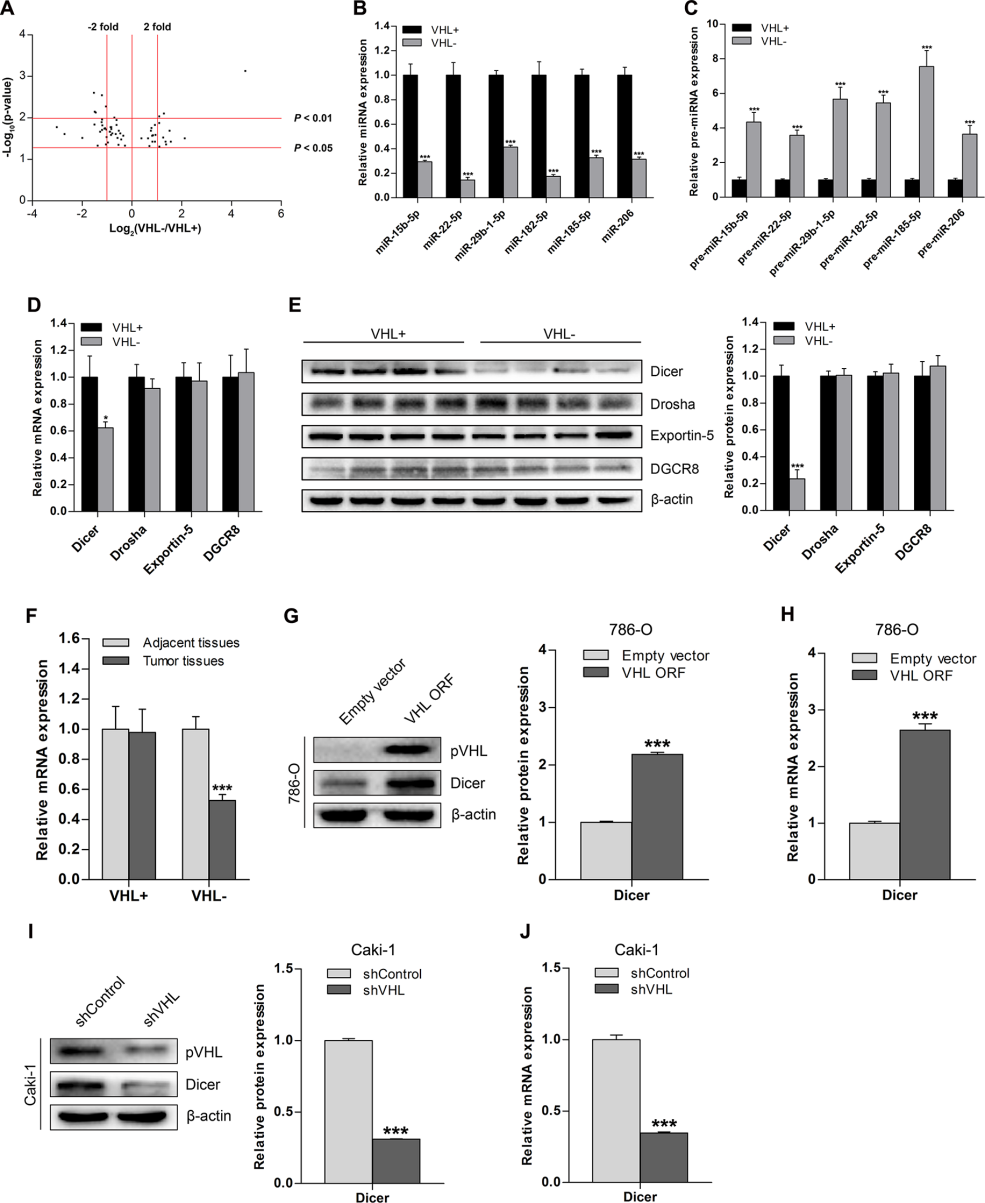
Downregulation of Dicer and its global effect on miRNA in ccRCC is VHL-dependent (**A**) Volcano plot of 61 mature miRNAs for which there were significant differences in expression between 8 pairs of VHL− and VHL+ ccRCC tissue samples as assessed by microarray analysis. (**B** and **C**) qRT-PCR analysis of expression levels of representative downregulated mature miRNAs and corresponding upregulated precursor miRNAs in VHL− ccRCCs (*n* = 36) compared to VHL+ ccRCCs (*n* = 36). (**D**) mRNA levels of miRNA biogenesis components in VHL− ccRCCs (*n* = 36) compared to VHL+ ccRCCs (*n* = 36). (**E**) Representative immunoblots and quantification of protein levels of miRNA biogenesis components in VHL− ccRCCs (*n* = 12) compared with VHL+ ccRCCs (*n* = 12). (**F**) Dicer mRNA levels in VHL− ccRCCs (*n* = 36) and VHL+ ccRCCs (*n* = 36) compared to their corresponding adjacent normal tissues (*n* = 36 for both groups). (**G**) Representative immunoblots and quantification of Dicer protein levels in VHL-deficient and pVHL-restored 786-O cells. (**H**) Dicer mRNA levels in VHL-deficient and pVHL-restored 786-O cells. (**I**) Representative immunoblots and quantification of Dicer protein levels in wild-type VHL and VHL-knockdown Caki-1 cells. (**J**) Dicer mRNA levels in wild-type VHL and VHL-knockdown Caki-1 cells. VHL+ denotes wild-type VHL, and VHL− denotes VHL-deficient. Data are presented as mean ± SEM (*n* ≥ 3). *p < 0.05; **p < 0.01; ***p < 0.001 (Student's *t*-test).

A total of 182 ccRCC tissue samples were tested for VHL gene status; 135 had a VHL mutation, 11 had methylated VHL promoters, and 36 had neither of these characteristics. Both VHL mutation and methylation were categorized as VHL-deficient, and the absence of these deficiencies was defined as wild-type VHL. To investigate VHL-dependent miRNAs in ccRCC, we performed miRNA microarray analysis on eight VHL-deficient ccRCC tissue samples and compared the results to those obtained from eight wild-type VHL ccRCC tissue samples. Volcano plot analysis of the microarray data revealed significant differences in the expression of 61 miRNAs (*p* < 0.05) between the VHL-deficient group and wild-type VHL group; 39 of these 61 miRNAs were downregulated in the VHL-deficient group (Figure 1A). Additionally, 20 of 31 differentially regulated miRNAs with at least a 2-fold expression difference at significance of *p* < 0.05 and 6 of 9 differentially regulated miRNAs at significance of *p* < 0.01 were also downregulated in the VHL-deficient group (Figure 1A). To confirm the microarray results, we analyzed the expression of 6 representative downregulated miRNAs in 36 VHL-deficient and 36 wild-type VHL ccRCCs using quantitative real-time PCR (qRT-PCR); as expected, all 6 mature miRNAs were downregulated in the VHL-deficient group (Figure 1B). Additionally, levels of the precursor species for all 6 miRNAs were increased in VHL-deficient ccRCCs (Figure 1C), suggesting that VHL deficiency impairs miRNA biogenesis.

Therefore, we examined the expression of Dicer and other key miRNA processing enzymes in VHL-deficient and wild-type VHL ccRCCs. Dicer mRNA and protein levels were lower in VHL-deficient ccRCCs than in wild-type VHL ccRCCs, but the expression of other factors did not differ between the two groups (Figure 1D and 1E). Dicer was also downregulated in VHL-deficient ccRCCs compared to adjacent normal tissues, but there was no difference in Dicer expression between wild-type VHL ccRCCs and adjacent normal tissues (Figure 1F).

We then examined changes in Dicer expression after increasing pVHL levels in a VHL-deficient ccRCC cell line (786-O) and decreasing pVHL levels in a wild-type VHL ccRCC cell line (Caki-1). Dicer protein and mRNA levels were higher in pVHL-transfected 786-O cells compared to cells transfected with the empty vector (Figure 1G and 1H). Knockdown of pVHL in Caki-1 cells reduced Dicer protein and mRNA levels compared to wild-type control Caki-1 cells (Figure 1I and 1J). Taken together, these results indicate that VHL deficiency downregulates Dicer expression in ccRCC.

### Dicer reduces HIF-2α expression in VHL-deficient ccRCCs

**Figure 2:**
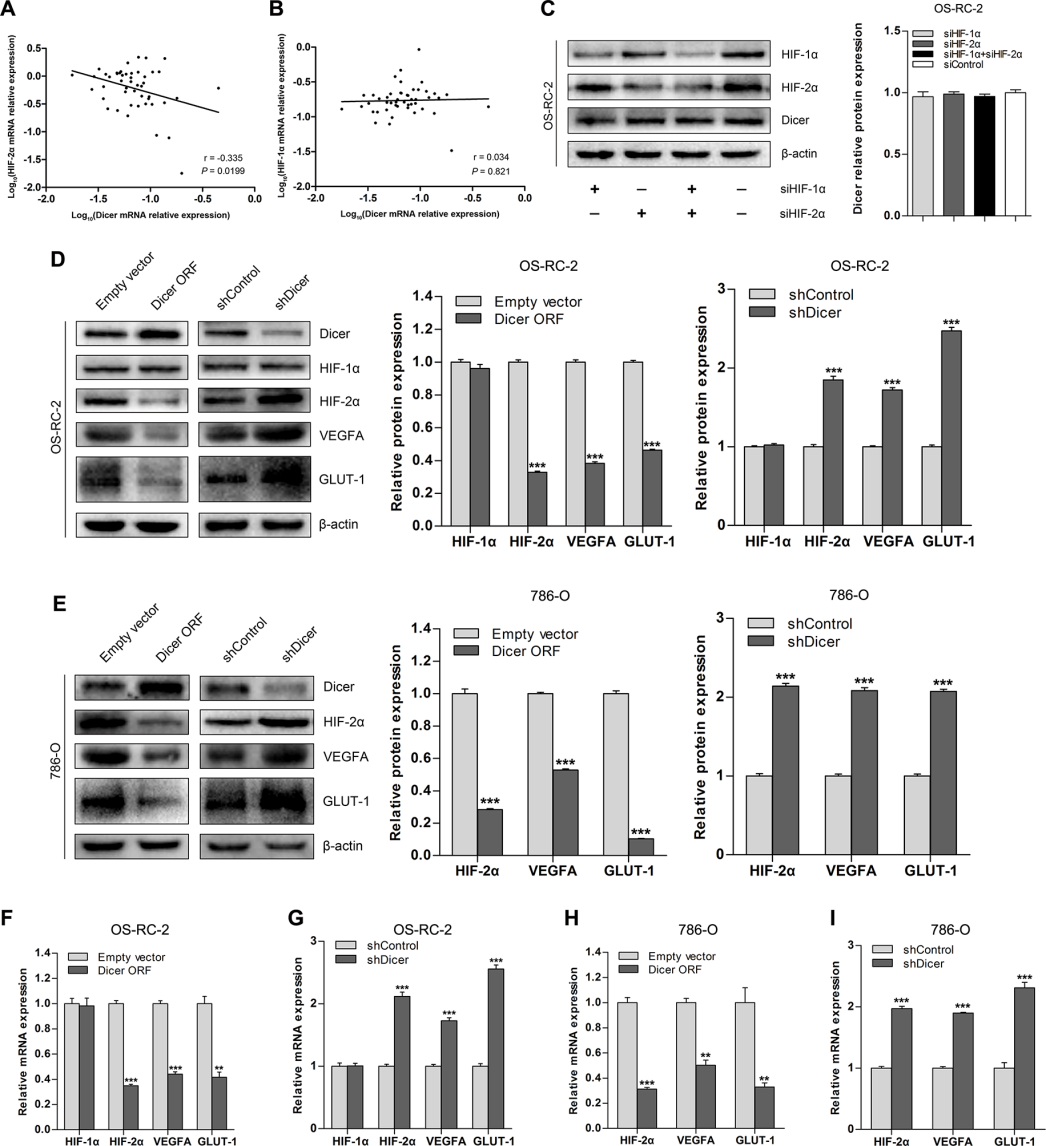
Dicer downregulates HIF-2α expression in VHL-deficient ccRCCs (**A** and **B**) Relationship between HIF-2α and HIF-1α mRNA levels and Dicer mRNA levels in ccRCCs (*n* = 48). (**C**) Representative immunoblots and quantification of Dicer protein levels after knockdown of HIF-1α, HIF-2α, or both in OS-RC-2 cells. (**D**) Representative immunoblots and quantification of HIF-1α, HIF-2α, VEGFA, and GLUT-1 protein levels after Dicer ectopic expression (Dicer ORF) or Dicer knockdown (shDicer) in OS-RC-2 cells. (**E**) Representative immunoblots and quantification of HIF-2α, VEGFA, and GLUT-1 protein levels after Dicer ectopic expression (Dicer ORF) or Dicer knockdown (shDicer) in 786-O cells. (**F** and **G**) HIF-1α, HIF-2α, VEGFA, and GLUT-1 mRNA levels after Dicer ectopic expression (Dicer ORF) or Dicer knockdown (shDicer) in OS-RC-2 cells. (**H** and **I**) HIF-2α, VEGFA, and GLUT-1 mRNA levels after Dicer ectopic expression (Dicer ORF) or Dicer knockdown (shDicer) in 786-O cells. ORF = open reading frame. Data are presented as mean ± SEM (*n* ≥ 3). *p < 0.05; **p < 0.01; ***p < 0.001 (Student's *t*-test).

Given that VHL is a critical regulator of HIF-α, we investigated whether downregulation of Dicer under VHL deficiency is mediated by HIF. We analyzed Dicer, HIF- 1α, and HIF-2α mRNA levels in ccRCC tissue samples (*n* = 48); there was an inverse correlation between Dicer and HIF-2α levels (Figure 2A), but not between Dicer and HIF-1α levels (Figure 2B). To determine whether Dicer expression is HIF-dependent in ccRCC cells, we examined a VHL-deficient ccRCC cell line, OS-RC-2, that stably expresses both HIF-1α and HIF- 2α. Dicer expression did not change in these cells after siRNA-induced decreases in the levels of HIF-1α, HIF- 2α, or both (Figure 2C), indicating that Dicer expression is not affected by cellular HIF-α status.

Because Dicer levels correlated with HIF-2α levels in ccRCC tissue samples, we tested whether the regulation of HIF-2α is Dicer-dependent. Dicer overexpression in OS-RC-2 cells reduced HIF-2α mRNA and protein levels, as well as levels of the downstream target genes vascular endothelial growth factor A (VEGFA) and glucose transporter 1 (GLUT-1) (Figure 2D and 2F). However, HIF-1α levels were not affected by Dicer overexpression (Figure 2D and 2F). Dicer knockdown following transfection of short hairpin Dicer (shDicer) RNA into OS-RC-2 cells upregulated HIF-2α, VEGFA, and GLUT-1 expression, but did not affect HIF-1α expression (Figure 2D and 2G). To determine whether these findings extend to the VHL-deficient ccRCC cell line stably expressing only HIF-2α, we also altered Dicer expression in 786-O cells. Overexpression of Dicer downregulated, and knockdown of Dicer upregulated, levels of HIF-2α and its downstream target genes in 786-O cells (Figure 2E, 2H, and 2I). Finally, we performed the same overexpression and knockdown experiment in 786-O cells with restored pVHL levels (786-O-pVHL). HIF-2α expression did not change when Dicer was overexpressed or depleted (Figure S1), suggesting that Dicer-dependent regulation of HIF-2α may not occur in wild-type VHL ccRCC cells. Together, these studies show that Dicer suppresses HIF-2α expression in VHL-deficient ccRCCs.

### HIF-2α is a direct target of Dicer-dependent miR-182-5p

**Figure 3:**
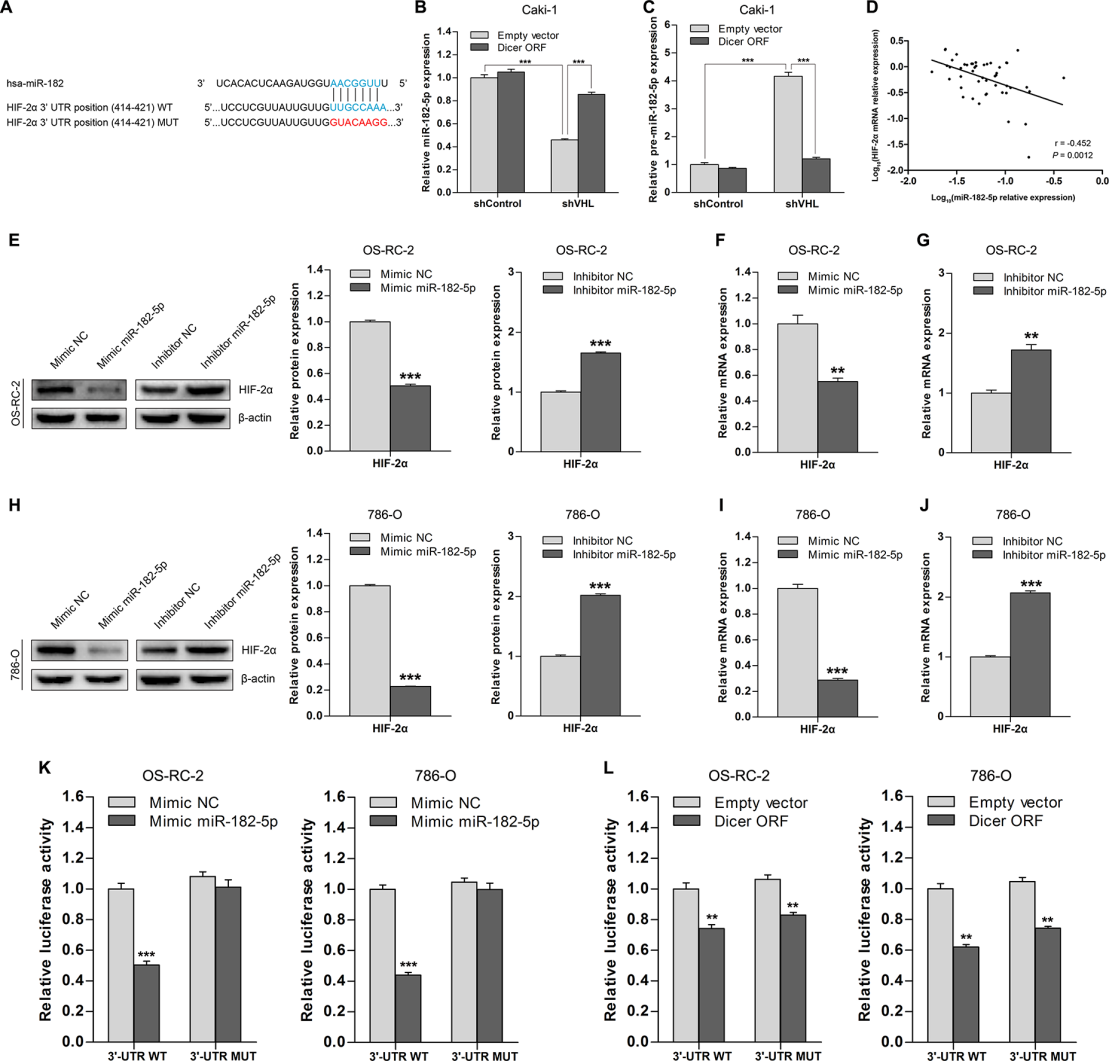
HIF-2α is a direct target of Dicer-dependent miR-182-5p (**A**) Schematic representation of the potential binding site of miR-182-5p in the HIF-2α 3′-UTR and its wild type (WT) or mutated (MUT) sequence within the luciferase reporter vector. (**B** and **C**) Levels of mature miR-182-5p and precursor miR-182-5p after Dicer ectopic expression (Dicer ORF) in VHL wild-type and VHL knockdown Caki-1 cells. (**D**) Association between HIF-2α mRNA levels and miR-182-5p levels in ccRCCs (*n* = 48). (**E**) Representative immunoblots and quantification of HIF-2α protein levels after miR-182-5p ectopic expression (mimic miR-182-5p) or miR-182-5p inhibition (inhibitor miR-182-5p) in OS-RC-2 cells. (**F** and **G**) HIF-2α mRNA levels after miR-182-5p ectopic expression (mimic miR-182-5p) or miR-182-5p inhibition (inhibitor miR-182-5p) in OS-RC-2 cells. (**H**) Representative immunoblots and quantification of HIF-2α protein levels after miR-182-5p ectopic expression (mimic miR-182-5p) or miR-182-5p inhibition (inhibitor miR-182-5p) in 786-O cells. (**I** and **J**) HIF-2α mRNA levels after miR-182-5p ectopic expression (mimic miR-182-5p) or miR-182-5p inhibition (inhibitor miR-182-5p) in 786-O cells. (**K**) Luciferase activity in OS-RC-2 and 786-O cells transfected with HIF-2α 3′-UTR luciferase reporter vectors with intact or mutated seed sequences in the presence of miR-182-5p mimics or NC, respectively. (**L**) Luciferase activity in OS-RC-2 and 786-O cells transfected with HIF-2α 3′-UTR luciferase reporter vectors with intact or mutated seed sequences in the presence of Dicer ORF or Empty vector, respectively. ORF = open reading frame. Data are presented as mean ± SEM (*n* ≥ 3). *p < 0.05; **p < 0.01; ***p < 0.001 (Student's *t*-test).

Because it is a key miRNA processing enzyme, Dicer likely suppresses HIF-2α expression in VHL-deficient ccRCCs via miRNA targeting. To test this hypothesis, we performed *in silico* analysis [16, 17] to identify specific miRNAs among those downregulated in VHL-deficient ccRCCs that might target HIF- 2α. miR-182-5p was present in eight evolutionarily conserved binding sites in the HIF-2α 3′-untranslated region (3′- UTR) sequence (Figure 3A). Notably, mature miR- 182-5p levels were decreased, and miR-182-5p precursor levels increased, in pVHL knockdown Caki-1 cells compared to wild-type VHL cells (Figure 3B and 3C). Ectopic Dicer expression in pVHL knockdown Caki- 1 cells rescued mature miR- 182-5p levels (Figure 3B) and restored lower levels of the precursor form (Figure 3C). Therefore, VHL-induced changes in miR-182-5p levels were Dicer-dependent.

Next, we analyzed the relationship between miR-182-5p and HIF-2α mRNA levels in ccRCC tissue samples (*n* = 48); miR-182-5p levels were inversely correlated with HIF-2α levels (Figure 3D). To determine whether miR-182-5p could regulate HIF-2α, we transfected miR-182-5p mimics into OS-RC-2 and 786-O cells, which reduced HIF- 2α protein and mRNA levels compared to control groups (Figure 3E–3J). Furthermore, transfection of a miR-182-5p inhibitor into OS-RC-2 and 786-O cells increased HIF- 2α protein and mRNA levels compared to control group cells (Figure 3E–3J). These results indicate that miR-182-5p inhibits HIF-2α expression in ccRCC cells.

To determine whether miR-182-5p directly targets the potential binding sites in the HIF-2α 3′-UTR, we generated luciferase reporter vectors in which the 3′-UTR sequence of HIF-2α, containing either wild-type or mutant miR-182- 5p binding sites (Figure 3A), was cloned downstream of the luciferase open reading frame. The reporter vectors were co-transfected with miR-182-5p mimics or a Dicer plasmid in OS-RC-2 and 786-O cells. Overexpression of miR-182-5p inhibited wild-type reporter activity, but not mutant reporter activity (Figure 3K), indicating that miR-182-5p directly targets HIF-2α. Notably, overexpression of Dicer inhibited activity of both the wild-type and mutant reporters (Figure 3L), suggesting that the ability of miR-182-5p to inhibit HIF-2α is Dicer-dependent, and other Dicer-dependent miRNAs may similarly regulate HIF-2α. Overall, these results indicate that HIF-2α is a direct target of Dicer-dependent miR-182-5p.

### Dicer suppresses tumor growth and angiogenesis *in vitro* by targeting HIF-2α in VHL-deficient ccRCCs

**Figure 4:**
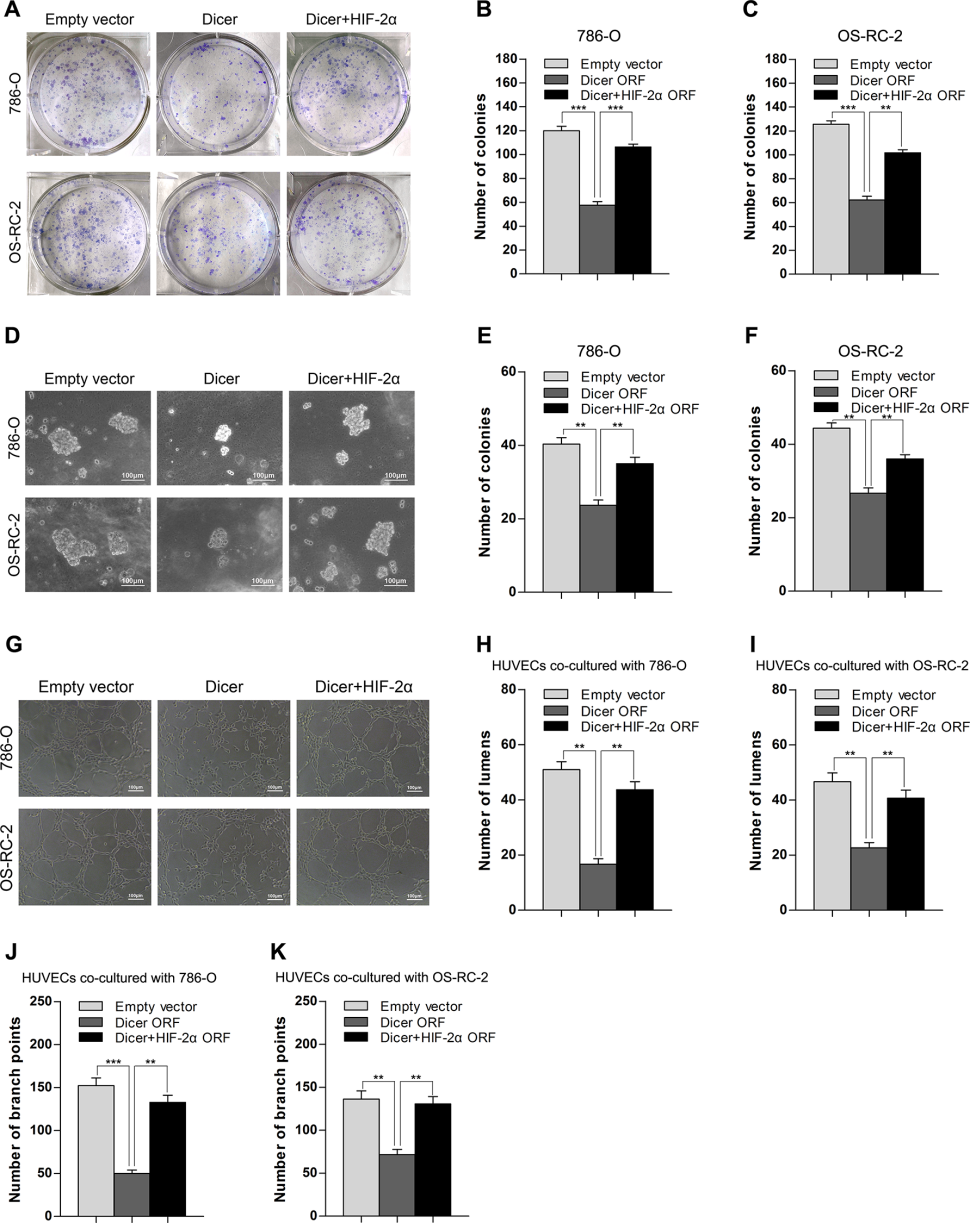
Overexpression of Dicer suppresses the tumor growth and angiogenesis *in vitro* by reducing HIF-2α in VHL-deficient ccRCCs (**A**–**C**) Representative images and quantification of an anchorage-dependent colony formation assay after Dicer ectopic expression (Dicer ORF) or Dicer ectopic expression plus HIF-2α rescue (Dicer + HIF-2α ORF) in 786-O and OS-RC-2 cells. (**D**–**F**) Representative images and quantification of anchorage-independent growth in a soft agar assay after Dicer ectopic expression (Dicer ORF) or Dicer ectopic expression plus HIF-2α rescue (Dicer + HIF-2α ORF) in 786-O and OS-RC-2 cells. (**G**–**K**) Representative images and quantification of a tube formation assay (number of lumens or branch points) in HUVECs co-cultured with 786-O or OS-RC-2 cells after Dicer ectopic expression (Dicer ORF) or Dicer ectopic expression plus HIF-2α rescue (Dicer + HIF-2α ORF). ORF denotes open reading frame. Data are presented as mean ± SEM (*n* ≥ 3). *p < 0.05; **p < 0.01; ***p < 0.001 (Student's *t*-test).

Next, we investigated whether Dicer overexpression could suppress the malignant phenotype in ccRCC by reducing HIF-2α expression in VHL-deficient ccRCC cells. In a colony formation assay, ectopic expression of Dicer attenuated anchorage-dependent growth of 786-O and OS-RC-2 cells compared to those transfected with empty vectors (Figure 4A–4C). In addition, a soft agar assay also indicated that ectopic Dicer expression reduced anchorage-independent growth of 786-O and OS-RC-2 cells (Figure 4D–4F). Tube formation, an important part of angiogenesis, was suppressed in HUVEC cells when they were co-cultured with either 786-O or OS-RC-2 cells expressing ectopic Dicer (Figure 4G–4K). To determine whether HIF-2α is involved in these effects, an ectopic HIF-2α open reading frame was introduced into 786-O or OS-RC-2 cells with stable overexpression of Dicer. Restoration of HIF-2α expression reversed the Dicer-induced phenotypes in these assays (Figure 4A–4K). These results indicate that Dicer suppresses tumor growth and angiogenesis *in vitro* in VHL-deficient ccRCCs at least in part by reducing HIF-2α expression.

### Overexpression of Dicer attenuates *in vivo* tumor growth in a VHL-deficient ccRCC model

**Figure 5:**
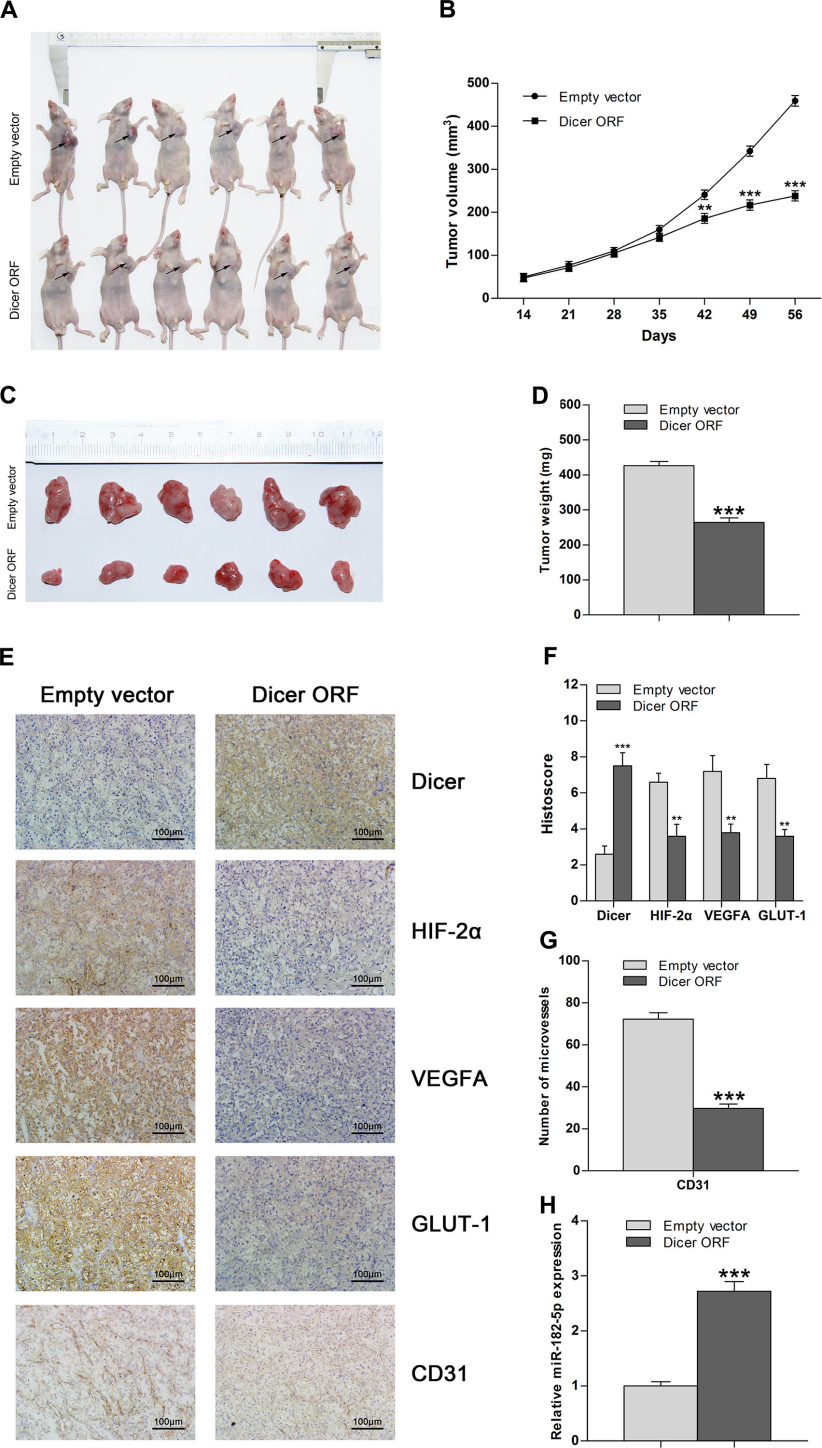
Overexpression of Dicer attenuates tumor growth in VHL-deficient ccRCC *in vivo* (**A**) Representative images of athymic nude mice with xenograft tumors of different sizes derived from subcutaneous implantation of 786-O cells treated with Dicer ORF or empty vector. (**B**) Xenograft tumor growth curve for the Dicer ORF-treated group compared to the empty vector-treated group (*n* = 10 per group). (**C**) Representative images of xenograft tumor burdens excised from nude mice 56 d after subcutaneous implantation of 786-O cells treated with Dicer ORF or empty vector. (**D**) Aggregate tumor weight of excised tumor burdens of the Dicer ORF-treated group compared to the empty vector-treated group (*n* = 10 per group). (**E**) Representative images of immunohistochemical staining of Dicer, HIF-2α, VEGFA, GLUT-1, and CD31 in tumors from the Dicer ORF-treated group and the empty vector-treated group. (**F**) Histoscores for Dicer, HIF-2α, VEGFA, and GLUT-1 in the Dicer ORF-treated group compared to the empty vector-treated group (*n* = 10 per group). (**G**) Intratumoral vessel count obtained by staining for CD31 in the Dicer ORF-treated group compared with that in empty vector-treated group (*n* = 10 per group). (**H**) miR-182-5p levels in the Dicer ORF-treated group compared with that in the empty vector-treated group (*n* = 10 per group). ORF = open reading frame. Data are presented as mean ± SEM (*n* ≥ 3). *p < 0.05; **p < 0.01; ***p < 0.001 (Student's *t*-test).

To investigate whether increased Dicer expression affects tumor growth of VHL-deficient ccRCC *in vivo*, we subcutaneously implanted immunocompromised nude mice with 786-O cells stably expressing either ectopic Dicer or empty vector. Xenograft tumor volumes (Figure 5A and 5B) and the aggregate weight of excised tumors (Figure 5C and 5D) were lower in the group expressing ectopic Dicer 42 and 56 days after implantation, respectively, as compared to the control group. This indicates that increased expression of Dicer inhibits xenograft tumor growth. Immunohistochemical staining confirmed that Dicer levels increased, whereas expression of HIF-2α and downstream genes and CD31-positive mean vessel densities decreased, in the group expressing ectopic Dicer (Figure 5E–5G). Finally, real-time PCR showed that miR-182-5p levels also increased in the ectopic Dicer expression group (Figure 5H). These results are consistent with the *in vitro* findings that overexpression of Dicer suppresses tumor growth and angiogenesis in VHL-deficient ccRCCs by reducing HIF- 2α expression.

### Reduced Dicer levels predict poor survival in VHL-deficient ccRCC patients

**Figure 6:**
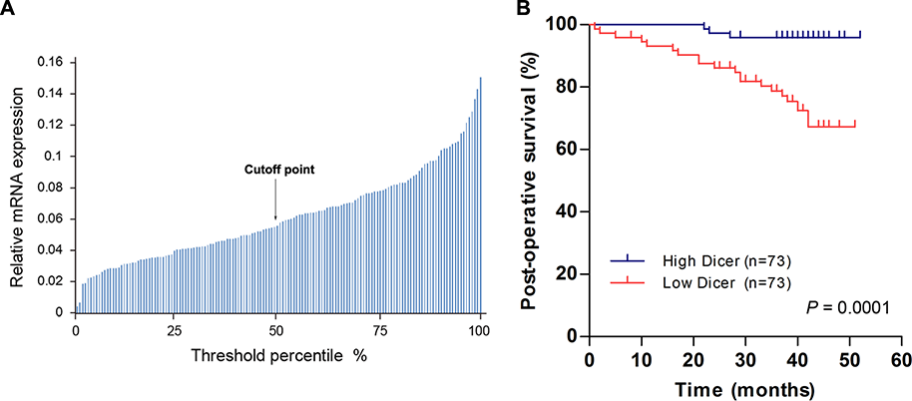
Lower Dicer levels predict shorter post-operative survival in VHL-deficient ccRCC patients (**A**) Dicer mRNA levels in 146 ccRCCs with VHL mutations or methylation of VHL promoters are shown; the median Dicer mRNA level served as the cutoff point between “High Dicer” and “Low Dicer” groups. (**B**) Kaplan–Meier survival curves for VHL-deficient ccRCC patients patients with high (*n* = 73) and low (*n* = 73) Dicer mRNA levels (*p* = 0.0001; log-rank test).

To investigate whether Dicer expression levels correlate with VHL-deficient ccRCC patient survival, we followed up with 146 ccRCC patients with VHL mutations or promoter methylation for 1–52 months (median, 39 months). After analyzing Dicer mRNA levels in these 146 ccRCC tissue samples, we selected the median Dicer level as the cutoff point between the high and low Dicer groups (*n* = 73 per group; Figure 6A). Kaplan–Meier analysis demonstrated that patients with low Dicer levels had poorer post-operative survival rates than those with high Dicer levels (*p* = 0.0001; Figure 6B), and univariate analysis indicated that low Dicer levels were a prognostic factor (Hazard ratio, HR = 7.654; 95% confidence interval, CI = 2.259–25.931; *p* = 0.001; Table 1). Multivariate analysis also indicated that low Dicer levels were an independent prognostic factor (HR = 6.088; 95% CI = 1.701–21.787; *p* = 0.005) separate from other clinicopathological parameters, such as gender, age, body mass index (BMI), tumor size, overall tumor, node, and metastasis (TNM) staging, and Fuhrman grade (Table 1). Taken together, these results indicate that reduced Dicer levels predict poor survival in VHL-deficient ccRCC patients.

**Table 1:** Univariate and multivariate analysis of clinicopathologic parameters and Dicer mRNA levels with regard to post-operative survival

Variables	Univariate analysis			Multivariate analysis		
	HR	95% CI	*P*-value	HR	95% CI	*P*-value
Gender						
Male	1			1		
Female	0.459	0.155–1.358	0.159	0.926	0.281–3.047	0.899
Age (years)						
< 60	1			1		
≥ 60	3.977	1.666–9.494	0.002	3.765	1.472–9.635	0.006
BMI						
< 25	1			1		
≥ 25	0.882	0.381–2.043	0.770	0.775	0.320–1.881	0.574
Tumor size (cm)						
≤ 7	1			1		
> 7	7.054	3.011–16.523	< 0.001	2.981	1.040–8.544	0.042
Overall TNM staging						
I + II	1			1		
III + IV	13.240	5.590–31.359	< 0.001	4.185	1.521–11.514	0.006
Fuhrman grade						
1 + 2	1			1		
3 + 4	5.592	2.384–13.117	< 0.001	1.736	0.658–4.582	0.265
Dicer levels						
High	1			1		
Low	7.654	2.259–25.931	0.001	6.088	1.701–21.787	0.005

TNM staging: TNM stage grouping was assigned according to the 2009 TNM staging classification system. Abbreviation: BMI, body mass index; HR, hazard ratio; CI, confidence interval.

## DISCUSSION

Loss of pVHL activity occurs in the majority of sporadic ccRCCs and results in accumulation of HIF subunits and transactivation of HIF-responsive genes [7, 18]. miRNAs are globally downregulated in ccRCCs [12, 13, 19–21], and specific roles for various miRNAs in ccRCCs have been reported [22, 23]. The pVHL/HIF pathway and its regulation by miRNAs play important roles in the development and progression of ccRCCs. We previously reported that Dicer, the key enzyme for processing and generating mature miRNAs, is downregulated in ccRCCs [15], but the cause of this downregulation is unknown. Here, we demonstrated that VHL deficiency downregulated Dicer expression, led to global downregulation of mature miRNAs, and resulted in the accumulation of precursor miRNAs. Dicer was only downregulated in VHL-deficient ccRCCs, and not in adjacent normal tissues or in wild-type VHL ccRCCs or their matched normal tissues. Given that pVHL loss is a common phenomenon in ccRCC, VHL mutations and methylation might largely explain the downregulation of Dicer and resultant miRNA biogenesis defects in this disease.

Our data suggest that the regulation of Dicer expression by VHL occurs at least in part through a transcriptional mechanism, because Dicer mRNA and protein levels are both affected by VHL status. Given that *in silico* analysis showed no binding sequence for pVHL in the Dicer promoter region and that VHL-dependent regulation of Dicer was HIF-independent, additional intermediate mechanisms are likely involved in this process. Regulation of Dicer transcription has been studied extensively. For example. Levy *et al.* [24] reported that microphthalmia-associated transcription factor binds and activates a conserved regulatory element upstream of the Dicer transcriptional start site upon melanocyte differentiation, and Su *et al.* [25] demonstrated that TAp63 directly regulates Dicer transcription. pVHL regulates the activity of various transcription factors, including p53 [26, 27]; therefore, pVHL-induced inactivation of TAp63, which is a member of the p53 family, may account for reduced Dicer levels in VHL-deficient ccRCCs. Additionally, Ho *et al.* [28] reported downregulation of Dicer under hypoxic conditions, which might also contribute to reduced Dicer expression in ccRCC.

It has long been thought that HIF activity is controlled exclusively by pVHL-mediated protein degradation, and that increased HIF activity following pVHL loss in ccRCCs leads to tumor formation and pathologic angiogenesis [29]. However, growing evidence indicates that post-transcriptional regulation of HIFs by miRNAs also plays a role in these processes. HIF-1α activity is reduced by the miR-17-92 cluster [30] and by miR-519c [31], and HIF-2α is a direct target of miR-30c-2-3p, miR-30a-3p, miR-145, and miR-185 [28, 32, 33]. Here, we demonstrate that overexpression of Dicer reduced the expression of HIF-2α and its downstream genes. Because Dicer is the key enzyme for the production of mature miRNA, Dicer-dependent regulation of HIF-2α is likely due to changes in miRNA levels. We identified a new Dicer-dependent miRNA, miR-182-5p, which can directly target HIF-2α, and showed that miR-182-5p was downregulated in VHL-deficient ccRCCs, which increased the expression of HIF-2α. Downregulation of miR-182-5p plays an important role in the pathogenesis of ccRCC [34]. Moreover, our data demonstrate that miR-182-5p is not the only Dicer-dependent miRNA that regulates HIF-2α, which suggests that reduced Dicer activity and subsequent miRNA repression is important in maintaining elevated HIF-2α levels in VHL-deficient ccRCCs.

Although HIF-1α and HIF-2α are both subject to VHL-mediated degradation, HIF-2α expression is predominant in VHL-deficient ccRCCs [35]. Moreover, recent studies indicate that HIF-2α is the primary oncogene in ccRCC development, whereas HIF-1α shows characteristics of a suppressor gene [6, 8, 36, 37]. Our data demonstrate that, while HIF-2α expression was Dicer-dependent, HIF-1α regulation was not; Dicer may therefore affect VHL-deficient ccRCCs specifically by reducing HIF-2α expression. In this study, we found that Dicer overexpression attenuated growth and angiogenesis in VHL-deficient ccRCC cells, while restoration of HIF- 2α expression reversed these phenotypic traits *in vitro*. The tumor-suppressive effects of Dicer-induced HIF-2α reduction were further confirmed using a xenograft tumor model. Recent evidence has shown that Dicer is a haploinsufficient tumor suppressor gene [38] and that reducing Dicer levels, which results in impaired miRNA biogenesis, enhances oncogenic transformation through deregulation of target oncogenes [39]. HIF-2α is pivotal in promoting tumor aggressiveness and angiogenesis by trans-activating its downstream genes, including GLUT-1 and VEGFA [4, 40]. ccRCC growth depends on aerobic glycolysis for ATP production, and this reliance is largely due to GLUT-1 induction [41]. Pathological angiogenesis, in which VEGFA plays an important role, is another distinct characteristic of ccRCC [42]. Here, GLUT-1 and VEGFA were both downregulated by Dicer-induced HIF-2α repression, which could explain the phenotypic changes that occur when Dicer levels are altered in VHL-deficient ccRCCs.

Finally, our results confirmed that reduced Dicer expression was associated with poor survival in VHL-deficient ccRCC patients, consistent with the prognosis associated with reduced Dicer expression in lung, ovarian, and breast cancer patients [43–45]. This suggests that measuring Dicer levels in ccRCC patients may help to provide more accurate prognoses and guide decisions regarding the use of radical treatments.

In conclusion, we demonstrated that Dicer is regulated by pVHL and plays an important role in suppressing the tumorigenic phenotype by reducing expression of HIF-2α and its downstream genes in VHL- deficient ccRCCs. Reduced Dicer expression in VHL-deficient ccRCCs may be an independent prognostic factor for poor clinical outcome. Therefore, Dicer may potentially serve as a risk stratification marker and therapeutic target for managing ccRCC.

## MATERIALS AND METHODS

### Patients and tissue samples

This study was approved by the Ethics Committee of Chinese PLA General Hospital, and written informed consent was obtained from all included patients. A total of 182 ccRCC tissue samples, along with adjacent non-tumor tissue samples, were obtained from patients who underwent nephrectomy at the Urology Department of Chinese People's Liberation Army (PLA) General Hospital (Beijing, China) between 2009 and 2013. All specimens were immediately flash-frozen in liquid nitrogen after resection, and pathological diagnosis of ccRCC was confirmed by two senior pathologists. The TNM stages of the specimens were assigned according to the 2009 TNM staging classification system, and nuclear grades were determined using the Fuhrman nuclear grading system. Patients' characteristics are provided in Table 2. Follow-ups were conducted for a total of 146 ccRCC patients with VHL deficiency, and the median follow-up period was 39 months (range, 1–52 months); 20 patients died by the end of follow-ups.

**Table 2:** Patients' characteristics

Clinicopathological parameters	No. (%) (*n* = 182)
Gender	
Male	128 (70.3)
Female	54 (29.7)
Age (years)	
< 60	122 (67.0)
≥ 60	60 (33.0)
BMI	
< 25	77 (42.3)
≥ 25	105 (57.7)
Tumor size (cm)	
≤ 7	148 (81.3)
< 7	34 (18.7)
Overall TNM staging	
I	139 (76.4)
II	22 (12.1)
III	5 (2.7)
IV	16 (8.8)
Fuhrman grade	
1	37 (20.3)
2	102 (56.0)
3	37 (20.3)
4	6 (3.3)

TNM staging: TNM stage grouping was assigned according to the 2009 TNM staging classification system. Abbreviation: BMI, body mass index.

### VHL sequencing and VHL promoter methylation

Genomic DNA was extracted from tumor samples using the General AllGen Kit (ComWin Biotech, Beijing, China) according to the manufacturer's instructions. *VHL* exons were amplified from extracted DNA using PCR and were then sequenced by GENEWIZ Company (Beijing, China) as previously described [14]. Primers for the three *VHL* gene exons are listed in Supplementary Table S1. The methylation status of the *VHL* promoter was assessed using methylation-specific PCR following bisulfite treatment of extracted DNA using the EZ DNA Methylation^™^ Kit (Zymo Research) according to the manufacturer's instructions. Methylated- and unmethylated-specific primers for the *VHL* promoter are listed in Supplementary Table S2.

### Microarray analysis

High-quality total RNAs extracted from eight ccRCC VHL-deficient tissue samples and eight ccRCC tissue samples with wild-type VHL were analyzed for expression of 2,019 miRNAs (Sanger miRBase 20.0) at LC Sciences (Houston, TX) using their in-house developed μParaflo^™^ technology platform. Methods and data analysis were performed as previously described [46].

### Quantitative real-time PCR (qRT-PCR)

Total RNAs were extracted using TRIzol reagent (ComWin Biotech, Beijing, China). cDNA synthesis of regular genes was performed using a TransScript First-Strand cDNA Synthesis SuperMix Kit (TransGen Biotech, Beijing, China) according to the manufacturer's instructions. miRNAs were reverse-transcribed using specific stem-loop RT-PCR as previously described [47]. Afterward, qRT-PCR was performed using TransStart Green qPCR SuperMix (TransGen Biotech, Beijing, China) on an Applied Biosystems 7500 Detection System. Relative mRNA expressions were normalized to peptidylprolyl isomerase A (PPIA), and relative miRNA expressions were normalized to small nucleolar RNA U6, using the 2^−ΔΔCT^ method. The primers used for this set of experiments are listed in Supplementary Tables S3 and S4.

### Western blot analysis

Total proteins extracted from tissues or cells were fractionated using 10% SDS-polyacrylamide gel electrophoresis and transferred onto PVDF membranes (Millipore, Billerica, MA). After blocking with 5% non-fat milk for 1 h, the membranes were incubated with primary antibodies overnight at 4°C, followed by incubation with the corresponding secondary antibodies (ZSGB-BIO, Beijing, China) for 1 h at room temperature. Immunoreactive bands were visualized using an enhanced chemiluminescence detection system (Thermo, USA) and signal quantification was normalized to β-actin. The primary antibody sources are listed in Supplementary Table S5.

### Cell culture

786-O, OS-RC-2, and Caki-1 human ccRCC cell lines and human umbilical vein endothelial cells (HUVECs) were obtained from the National Platform of Experimental Cell Resources for Sci-Tech (Beijing, China). Cells were cultured in Dulbecco's modified Eagle's medium (DMEM; HyClone, USA) supplemented with 10% fetal bovine serum (Gibco, Grand Island, NY) and maintained at 37°C in an incubator with 5% CO_2_.

### Plasmids and viral vectors

The *VHL* open reading frame was cloned into a pWPI lentiviral vector (Addgene) to rescue wild-type VHL. For knockdown of VHL, annealed DNA oligonucleotides targeting *VHL* were cloned into a pLVTHM lentiviral vector (Addgene). For ectopic expression of Dicer, Dicer open reading frame was cloned into a pLVX-Puro lentiviral vector (Clonetech). To rescue HIF-2α expression, HIF-2α open reading frame was cloned into a pWPI lentiviral vector (Addgene). Viral particle generation and infection were performed as previously described [48].

### Transfection of small interfering RNAs (siRNAs) and miRNA mimics or inhibitors

siRNAs targeting HIF-1α and HIF-2α and miR-182-5p mimics or inhibitors were chemically synthesized by GenePharma (Shanghai, China). Transfection was conducted using Lipofectamine^®^ 2000 (Invitrogen, Carlsbad, CA) according to the manufacturer's instructions. The siRNA sequences of HIF-1α and HIF-2α are listed in Supplementary Table S6.

### Luciferase reporter assay

3′-UTRs of HIF-2α containing the miR-182-5p binding site with either the wild-type or mutated seed sequence were cloned into psiCHECK-2 dual-luciferase vectors (Promega, Madison, WI). To test the effect of miR-182-5p overexpression on luciferase activity, tumor cells were co-transfected with the wild-type or mutated 3′-UTR luciferase reporter and miR-182-5p mimics or controls. Tumor cells were co-transfected with the wild-type or mutated 3′-UTR luciferase reporters and Dicer plasmid or empty vector to test the effect of Dicer overexpression on luciferase activity. Firefly and *Renilla* luciferase activities were measured 48 h after transfection according to the Dual-Luciferase Assay Manual. The relative luciferase activity for each group was based on the *Renilla* luciferase signal normalized to the firefly luciferase signal.

### Colony formation assay

786-O and OS-RC-2 cells from the respective transfection groups were separately seeded into 6-well plates at a density of 1 × 10^3^ cells per well. Colonies were counted following methanol fixation and staining with 0.2% crystal violet after 14 d.

### Soft agar assay

786-O and OS-RC-2 cells from the respective transfection groups were suspended in 0.35% top agar and then plated into 6-well plates with 0.7% base agar at a density of 1 × 10^3^ cells per well. After incubation for 3–4 weeks, colony numbers were counted.

### Tube formation assay

Growth factor-reduced basement membrane extract (BME; Trevigen, Gaithersburg, MD) was added to each well of a 96-well plate and allowed to polymerize for 1 h at 37°C. HUVECs co-cultured with ccRCC cells from the respective transfection groups for 48 h were preincubated with serum-free DMEM for 1 h and then seeded into the BME-coated wells at a density of 3 × 10^4^ cells per well. After incubation for 8 h, the numbers of lumens and branch points in each well were counted.

### *In vivo* xenograft tumor growth assay

Animal experiments were approved by the Animal Ethical Committee of Chinese PLA General Hospital. Male BALB/c nude mice aged 4–6 weeks old were used to establish xenograft tumor models. A total of 1 × 10^7^ 786-O cells stably transfected with Dicer open reading frame or empty vector were subcutaneously injected into the armpits of nude mice. After tumor formation, tumor volume was measured every 7 d using the equation *a* × *b*^2^/2, where *a* and *b* are the length and the width, respectively, of the tumor. All mice were sacrificed 56 d after injection, and tumor burdens were harvested for tumor weight measurement and immunohistochemistry staining.

### Immunohistochemical staining

Immunohistochemical staining of the xenograft tumors was performed as previously described [49]. Staining results were interpreted by two independent pathologists who were blind to group information. For Dicer, HIF-2α, VEGFA, and GLUT-1, staining intensity was scored 0 (no staining), 1 (weak), 2 (moderate), or 3 (strong), and staining extent was scored 0 (no staining), 1 (1%–10%), 2 (11%–50%), 3 (51%–90%), or 4 (91%–100%). The product of these 2 scores was defined as the histoscore and was used for analysis. For CD31, staining results were quantified by counting the number of microvessels in selected fields. The primary antibody sources are listed in Supplementary Table S5.

### Statistical analysis

All data are presented as the mean ± SEM of at least three independent experiments and were analyzed using Student's *t*-test unless otherwise stated. A *p* value less than 0.05 was considered statistically significant.

## SUPPLEMENTARY MATERIALS TABLES AND FIGURE


