# Supplementary material for: Dicer suppresses the malignant phenotype in VHL-deficient clear cell renal cell carcinoma by inhibiting HIF-2α
Source: Oncotarget. 2016 Mar 1;7(14):18280–94. doi: 10.18632/oncotarget.7807 (PMC4951288; doi:10.18632/oncotarget.7807)
Supplement: Supplementary file 1 [file oncotarget-07-18280-s001.pdf]

Dicer suppresses the malignant phenotype in VHL-deficient clear cell renal cell carcinoma by inhibiting HIF-2α

Supplementary Materials

Supplementary Table S1: Primer sequences for three exons of VHL

Gene	Forward Primer	Reverse Primer
VHL-Exon 1	5′-AGCGCGTTCCATCCTCTAC-3′	5′-GAATGCTCTGACGCTTACGA-3′
VHL-Exon 2	5′-CTCCTGGGTTCACACCATTC-3′	5′-AGCCCAAAGTGCTTTTGAGA-3′
VHL-Exon 3	5′-CAGTGTCGCTTCATCCACAT-3′	5′-CAAAAATGCCACCACCTTCT-3′

Supplementary Table S2: Methylated and unmethylated-specific primer sequences for VHL promoter

Specificity	Forward Primer	Reverse Primer
Methylated	5′-GGAGGATTATTTGAATTTAGGAGTTC-3′	5′-TTAAAACAAAATCTCACTCTATCGC-3′
Unmethylated	5′-GGATTATTTGAATTTAGGAGTTTGA-3′	5′-TAAAACAAAATCTCACTCTATCACC-3′

Supplementary Table S3: qRT-PCR primer sequences for regular genes

Gene	Forward Primer	Reverse Primer
Dicer	5′-GTGCGAGAATTGCTTGAA-3′	5′-CACAGTGACTCTGACCTT-3′
Drosha	5′-CAATGAGACGAGAAGTAACG-3′	5′-GGTGGTAGCGGATATGAT-3′
Exportin-5	5′-TCACCTACAGACCAGAGT-3′	5′-ATCTTGATGATGGAGGAACA-3′
DGCR8	5′-GCCTCAGGTAGAAGAAGAA-3′	5′-TTAGAACCACTGCCTCAAT-3′
HIF-1α	5′-AGGCCGCTCAATTTATGAAT-3′	5′-TTTGGCAAGCATCCTGTACT-3′
HIF-2α	5′-TGGCGACATGATCTTTCTGTCA-3′	5′-ATGGTCGCAGGGATGAGTGA-3′
VEGFA	5′-GGCAGAATCATCACGAAGT-3′	5′-CACAGGATGGCTTGAAGAT-3′
GLUT-1	5′-TATGTGGAGCAACTGTGT-3′	5′-TGAAGTAGGTGAAGATGAAGA-3′
PPIA	5′-TCATCTGCACTGCCAAGACTG-3′	5′-CATGCCTTCTTTCACTTTGCC-3′
Precursor miR-15b-5p	5′-TACTGTAGCAGCACATCAT-3′	5′-AATAATGATTCGCATCTTGACT-3′
Precursor miR-22-5p	5′-CCGCAGTAGTTCTTCAGT-3′	5′-AGGGCAACAGTTCTTCAA-3′
Precursor miR-29b-1-5p	5′-GGTTTCATATGGTGGTTTAGAT-3′	5′-TTTCAAATGGTGCTAGACAAT-3′
Precursor miR-182-5p	5′-CACACTGGTGAGGTAACA-3′	5′-CCCATAGTTGGCAAGTCTA-3′
Precursor miR-185-5p	5′-TTGGAGAGAAAGGCAGTT-3′	5′-GGAAGGACCAGAGGAAAG-3′
Precursor miR-206	5′-CCACATGCTTCTTTATATCCC-3′	5′-CACACTTCCTTACATTCCATAG-3′

Abbreviation: PPIA, peptidylprolyl isomerase A

Supplementary Table S4: Stem-loop RT and qRT-PCR primer sequences for miRNAs

miRNA	Stem-loop RT primer	Forward primer	Reverse primer
miR-15b-5p	5′-CTCACAGTACGTTGGTATC CTTGTGATGTTCGATGCCATA TTGTACTGTGAGTGTAAACC-3′	5′-ACACTCCAGCTGGGT GGTAGCAGCACATCATGG-3′	5′-CTCACAGTACGTTGG TATCCTTGTG-3′
miR-22-5p	5′-CTCACAGTACGTTGGTATC CTTGTGATGTTCGATGCCATA TTGTACTGTGAGTAAAGCTT-3′	5′-ACACTCCAGCTGGGTG GAGTTCTTCAGTGGCAA-3′	5′-CTCACAGTACGTTGG TATCCTTGTG-3′
miR-29b-1-5p	5′-CTCACAGTACGTTGGTATC CTTGTGATGTTCGATGCCATA TTGTACTGTGAGTCTAAACC-3′	5′-ACACTCCAGCTGGGTG GGCTGGTTTCATATGGTGG-3′	5′-CTCACAGTACGTTGG TATCCTTGTG-3′
miR-182-5p	5′-CTCACAGTACGTTGGTATCC TTGTGATGTTCGATGCCATATTG TACTGTGAGAGTGTGAG-3′	5′-ACACTCCAGCTGGGTG GTTTGGCAATGGTAGAACT-3′	5′-CTCACAGTACGTTGG TATCCTTGTG-3′
miR-185-5p	5′-CTCACAGTACGTTGGTATCC TTGTGATGTTCGATGCCATATT GTACTGTGAGTCAGGAAC-3′	5′-ACACTCCAGCTGGGTG GTGGAGAGAAAGGCAGT-3′	5′-CTCACAGTACGTTGG TATCCTTGTG-3′
miR-206	5′-CTCACAGTACGTTGGTATC CTTGTGATGTTCGATGCCATA TTGTACTGTGAGCCACACAC-3′	5′-ACACTCCAGCTGGGTG GTGGAATGTAAGGAAGT-3′	5′-CTCACAGTACGTTGG TATCCTTGTG-3′
U6	5′-AACGCTTCACGAATTTGCGT-3′	5′-CTCGCTTCGGCAGCACA-3′	5′-AACGCTTCACGAAT TTGCGT-3′

Supplementary Table S5: Antibodies' information

Antibody	Species	Applications and dilutions	Source
Dicer	Mouse	WB (1:1000); IHC (1:100)	Abcam (ab14601)
Drosha	Rabbit	WB(1:1000)	Cell Signaling (3364)
Exportin-5	Rabbit	WB (1:1000)	Cell Signaling (12565)
DGCR8	Rabbit	WB (1:500)	Santa Cruz (sc-134567)
VHL	Mouse	WB (1:4000)	Abcam (ab140989)
HIF-1α	Mouse	WB (1:500)	Novus Biologicals (NB100-131)
HIF-2α	Mouse	WB (1:500); IHC (1:200)	Novus Biologicals (NB100-132)
VEGFA	Rabbit	WB (1:500); IHC (1:100)	Santa Cruz (sc-152)
GLUT-1	Rabbit	WB (1:1000); IHC (1:200)	Abcam (ab652)
CD31	Rabbit	IHC (1:200)	ImmunoWay (YT0752)
β-actin	Mouse	WB (1:3000)	ZSGB-BIO (TA-09)

Supplementary Table S6: siRNA sequences used in this study

Gene	Sense	Antisense
HIF-1α	5′-CUGAUGACCAGCAACUUGATT-3′	5′-UCAAGUUGCUGGUCAUCAGTT-3′
HIF-2α	5′-CAGCAUCUUUGAUAGCAGUTT-3′	5′-ACUGCUAUCAAAGAUGCUGTT-3′
Negative control	5′-UUCUCCGAACGUGUCACGUTT-3′	5′-ACGUGACACGUUCGGAGAATT-3′

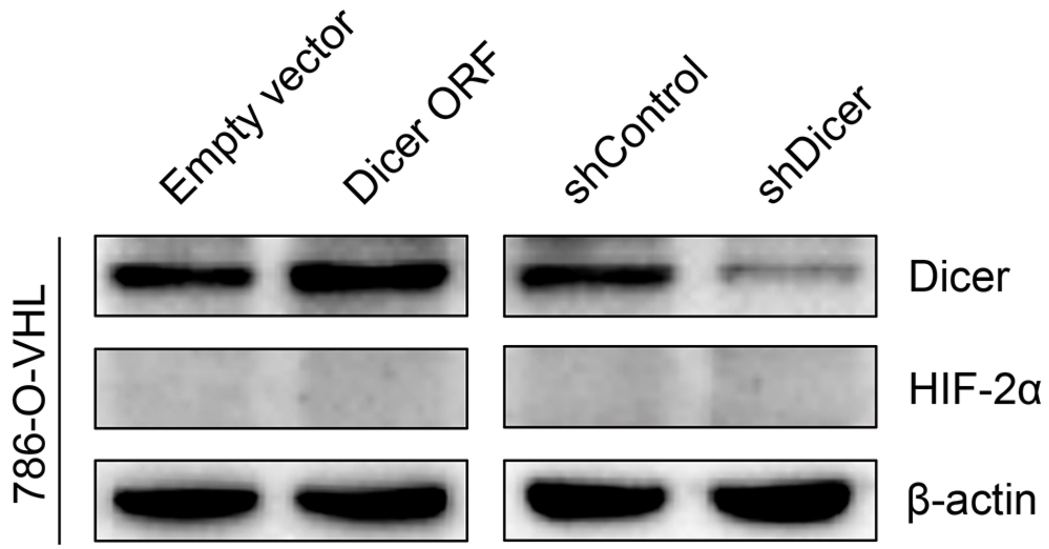

Supplementary Figure S1: Dicer does not affect HIF-2α expression in wild-type VHL ccRCCs
